# Memorial to John Hunter

**Published:** 1860-07

**Authors:** 


MEMORIAL TO JOHN HUNTER.—It is well known to our readers that a monument is about to be erected in Westminster Abbey to the memory of John Hunter, and the question may therefore be very properly asked, whether it would not be well for the physicians of this country to offer their co-operation to their British brethren in carrying out so laudable and worthy an object. Such a mark of courtesy would tend to encourage good feeling between the United States and England, and would, we doubt not, as in the case of Jenner, be promptly and cordially accepted. Besides, it would afford us an opportunity, on this side of the Atlantic, of acknowledging, in a somewhat substantial manner, our gratitude for the great and inestimable services conferred upon us by the writings and genius of the immortal man for whom the memorial is designed. Several of the State Medical Societies, as well as the American Medical Association, have already taken action in this matter, and we trust that Pennsylvania will not be slow in seconding their efforts. A subscription of one dollar for each physician would probably be a proper sum.

